# Unusual Metastatic Patterns of Wilms Tumor: A Case Series

**DOI:** 10.7759/cureus.54640

**Published:** 2024-02-21

**Authors:** Lily G Tagoe, Nana Yaa A Bonney, Emmanuella Amoako, Kokou H Amegan-Aho, Hafisatu Gbadamosi, Mary-Ann Dadzie, Catherine I Segbefia

**Affiliations:** 1 Child Health, Korle Bu Teaching Hospital, Accra, GHA; 2 Pediatrics and Child Health, University of Health and Allied Sciences, Ho, GHA; 3 Radiology, Korle Bu Teaching Hospital, Accra, GHA; 4 Radiotherapy and Nuclear Medicine, Korle Bu Teaching Hospital, Accra, GHA; 5 Child Health, University of Ghana Medical School, Accra, GHA

**Keywords:** wilms tumor, unusual sites, child, malignancy, relapse, metastases

## Abstract

Wilms tumor (WT) is the most common renal malignancy of childhood. The common metastatic sites are the lungs, liver, and lymph nodes, with brain and bone metastases occurring rarely. Metastatic disease can be present at initial diagnosis or may occur with relapse or disease progression. The majority of relapses in WT occur within the first two years post-treatment. Late relapses are rare. This article describes four cases of WT, each demonstrating an unusual site or timing of metastases. Case 1 presented primarily with jaw metastases, Case 2 presented with bone (vertebrae) and spinal metastases manifesting as paraplegia, at relapse one year after completion of treatment, Case 3 presented with isolated liver metastases four years after treatment completion, and Case 4 presented with brain metastases after six weeks of treatment abandonment. This case series demonstrates the varied pattern of metastases of WT and highlights the need for a high index of suspicion for WT among patients who present with unusual sites of tumor or for metastasis in those who present with neurologic symptoms during or after treatment.

## Introduction

Wilms tumor (WT) is the most common renal malignancy among children, accounting for about 90% of all renal neoplasms in the pediatric population [1,2]. It is also the most common abdominal malignancy in children in Ghana. WT characteristically occurs in younger childhood with a peak age of two to three years, with the majority (75-80%) of cases being diagnosed before age five [3,4]. The majority of WT cases are localized but about 12% present with metastatic disease at first presentation [5]. The most common site of metastases is the lung, accounting for about 80% of metastases [4,5]. Other less common sites are the liver and lymph nodes [6]. Bone and brain metastases are rare, occurring in approximately 1.3% and 1% of patients, respectively [7,8]. Outcomes are worse for metastatic compared to localized disease. Survival rates of WT globally are above 90% and about 75% for localized and metastatic disease, respectively [3]. Unlike other pediatric malignancies, the outcomes of WT have been duplicated in LMICs as well. In a study among children with WT in seven French African countries, the overall survival was 73% [9]. In a single center in Egypt, the overall survival was reported to be almost 95% [10].

About 15% of patients with WT will experience a relapse [11]. The most common sites of relapsed disease are the lungs and abdomen. The majority of relapses of WT occur within the first two years after treatment [7].

We describe four cases of WT, each demonstrating an unusual site or timing of metastases.

## Case presentation

Case 1

A three-year five-month-old male presented with a year’s history of progressive abdominal distension and an eight-month history of right-sided jaw swelling, associated with weight loss and bleeding from the intra-oral portion of the lesion. On examination, he had a right mandibular swelling measuring about 13 x 9 cm with intra-oral extension and dental anarchy. He also had gross abdominal distension with a palpable right flank mass. 

**Figure 1:**
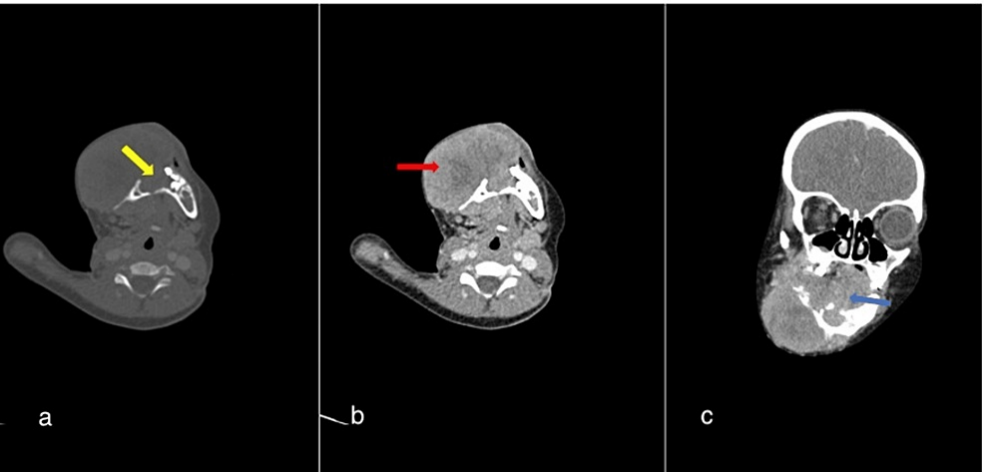
Contrast-enhanced CT scan of the head (a). Axial bone window (b and c). Axial and coronal reformatted soft tissue window. Large right hemimandibular mass with associated cortical destruction (yellow arrow), showing a large heterogeneously enhancing soft tissue mass, which is mainly exophytic (red arrow) with a smaller intra-oral extension (blue arrow) compressing the tongue.

Baseline computed tomography (CT) scan of the head showed a large expansile lytic destructive heterogeneously enhancing mass within the right hemimandible; it extended from the symphysis menti to the right angle of the mandible and was associated with a large externally exophytic soft tissue mass and a relatively smaller oral component. Resultant dental anarchy was present with a mild aggressive type periosteal reaction (Figure 1). The right cheek was laterally displaced by the mass.

**Figure 2:**
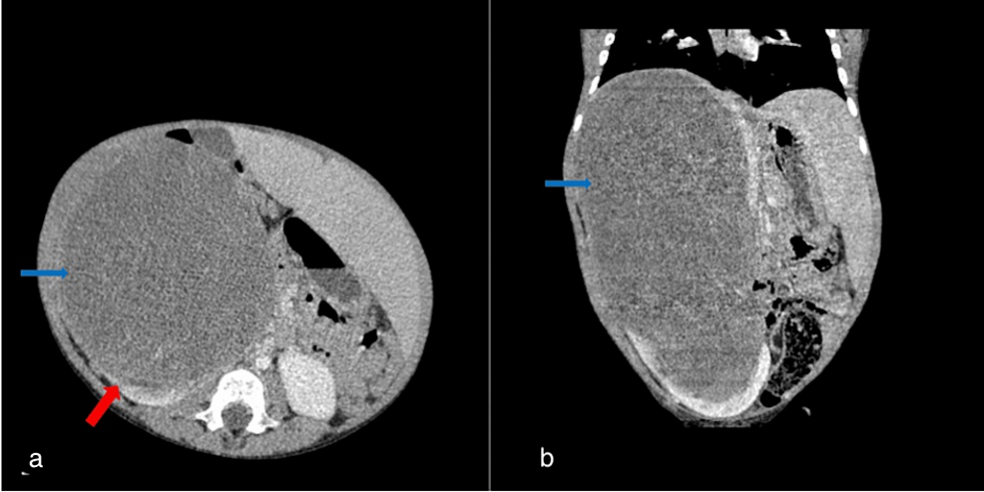
Axial and sagittal reformatted contrast CT of the abdomen and pelvis revealed a huge well-circumscribed mass (blue arrows) of renal origin evidenced by the claw sign (red arrow) (a and b). It extended to the level of the right hemidiaphragm with displacement of the right lobe of the liver and abdominal vessels.

The abdominopelvic CT scan showed a huge 21.0 CC, 12.2 AP, 10.9 TR cm right renal mass, shown in Figure 2. There were no lung metastases.

**Figure 3:**
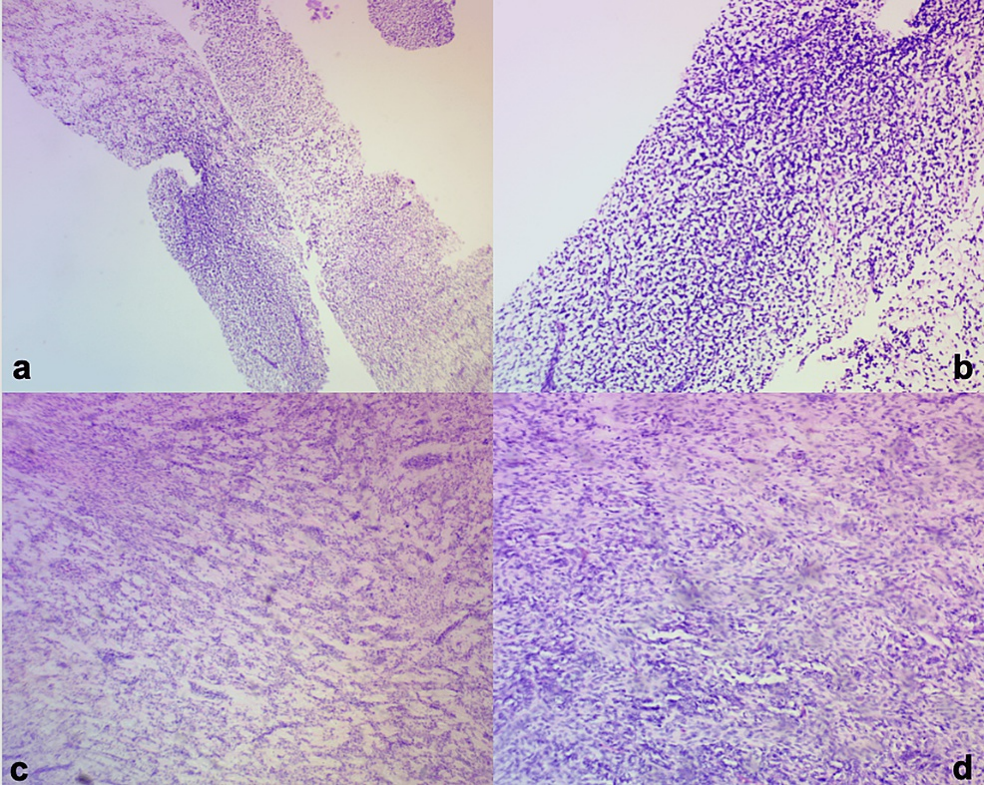
Photomicrographs showing histopathologic features of stromal predominant WT; (a) ×40 and (b) ×100, a core biopsy of the jaw mass; (c) ×40 and (d) ×100, sections from kidney.

Ultrasonography (USG)-guided biopsy of the renal mass was suggestive of a stromal component of WT but was inconclusive. Histopathology of a core biopsy of the jaw mass, however, showed a high-grade malignant neoplasm with hypo- and hypercellular areas separated by fibrocollagenous stroma (Figure 3). On immunohistochemistry, it stained strongly for Vimentin and WT-1 but there was no nuclear or cytoplasmic staining for SMA and Myogenin. The diagnosis made was right WT with jaw metastases. 

He received neoadjuvant chemotherapy with the International Society of Paediatric Oncology (SIOP) 2001 stage IV pre-operative treatment (Vincristine, Actinomycin D, and Doxorubicin), extended to nine weeks to improve respectability. 

He had a right radical nephrectomy thereafter with findings of a 25 x 10 cm right kidney with Gerota fascia firmly adherent to the diaphragm and right lobe of the liver. The mass was completely excised and assessed to be surgical stage II. On the histopathologic assessment of the specimen, there was no necrosis seen and the tumor was predominantly (95%) stromal with no evidence of anaplasia (Figure 3). Margins were not involved in the tumor. 

Post-surgery, he continued with adjuvant chemotherapy using the SIOP WT 2001 stage IV post-operative protocol for multiple inoperable metastases or incompletely resected disease, with alternating courses of Etoposide/Carboplatin and Cyclophosphamide/Doxorubicin for 34 weeks. 

After four courses of adjuvant chemotherapy, he underwent marginal resection of the right mandible with adjuvant application of Vincristine to the tumor bed. The findings were a fibrous-looking tumor causing thinning and buccal bone expansion with lingual perforation with the lesion extending to the lower border of the mandible and encapsulating the right neurovascular bundle. Histopathology revealed no residual tumor.

The patient is now eight months post-completion of treatment and is being followed up with regular physical examinations and abdominal ultrasound scans.

Case 2

A two-year-old female presented with a three-month history of a progressive left abdominal flank mass. At the presentation, she had no hypertension or hematuria. CT scan findings of the abdomen revealed a left renal mass. No pulmonary nodules were noted on the chest CT scan.

Diagnosis of localized left WT was made, and she was started on the SIOP pre-operative treatment protocol, which includes Actinomycin D and Vincristine. She received these for a total of six weeks and had surgery on her seventh week.

A left radical nephrectomy with descending colectomy was performed. Intra-operative findings were a 10 x 8 cm left kidney morbidly adhered to the posterior abdominal wall. Samples were sent for histopathology, which showed epithelial-type WT with clear resection margins. The pathological stage was I.

She received a total of eight weeks of Vincristine and Actinomycin D post-operatively. Follow-up in the first eight months with abdominopelvic USG and plain chest X-ray revealed no abnormal findings.

**Figure 4:**
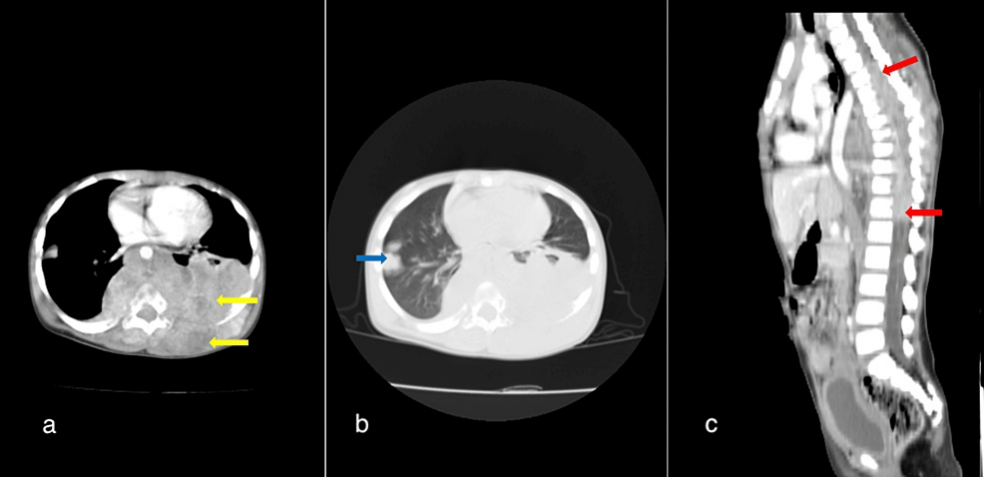
(a, b, and c) Contrast-enhanced CT of the chest and abdomen demonstrated an extensive lobulated and heterogeneously enhancing posterior mediastinal and pleural-based mass (yellow arrows), which displaced the thoracic aorta and heart anteriorly, also infiltrating the erecta spinae muscles, with bony destruction and extensive intraspinal extension ( red arrows). The child also had multiple bilateral lung nodules (blue arrow).

However, one year post-treatment, she presented with paraplegia in her lower limbs. Due to limited funding, magnetic resonance imaging (MRI) studies could not be conducted. She presented again months later when her mother noticed a mass protruding from her back. A chest and abdominopelvic CT revealed a huge posterior mediastinal and pleural-based mass, which was associated with extensive lytic destructive foci within the adjacent posterior ribs and thoracic vertebrae and extending into a long segment of the spinal canal (T4 to L1 level) where the cord was presumably compressed. The tumor shown also extended into both recta spinae and intercostal muscles. Additionally, several bilateral pulmonary nodules were present (Figure 4). 

Biopsy was taken with histopathology and IHC performed that confirmed metastatic WT. Parents were counseled on palliative care due to the extent of the disease.

Case 3

A 13-year-old female presented with a month’s history of abdominal pain and mass. She was initially diagnosed with localized left WT in April 2017 at age eight. She received neoadjuvant chemotherapy for four weeks with Vincristine and Actinomycin D, after which she had left radical nephrectomy. The surgical stage was III. Histopathology revealed a triphasic pattern with 90% epithelial and the rest stromal and blastemal. Pathological stage was I. She received adjuvant chemotherapy using the SIOP 2001 stage III intermediate risk protocol and received 27 weeks of Actinomycin D, Vincristine, and Doxorubicin. She also underwent flank radiotherapy to a dose of 10.8 Gy in six fractions. 

**Figure 5:**
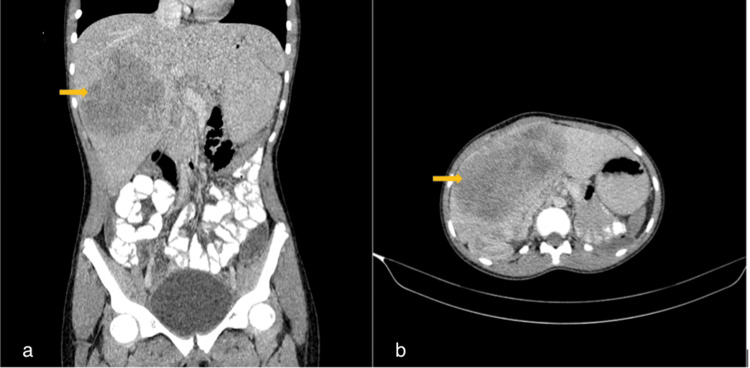
(a and b) Coronal reformatted axial contrast-enhanced abdominopelvic CT scan images. Demonstrating a large well-circumscribed heterogeneously enhancing mass in segments IVa, IVb, V, VI, and portions of VIII of the liver. Exerts mass effect on the right and main portal vein. No tumor thrombus or ascites present. No lung nodules present.

Post-treatment, she remained stable and was followed up with routine abdominal ultrasound scans. Four years post-treatment, in December 2021, she presented with right hypochondriac pain and swelling. Abdominal USG done showed a large heterogenous hepatic mass. A follow-up abdominopelvic CT scan revealed a hepatic mass centered on the right hepatic lobe, involving segments IV, V, VII, and VIII (Figure 5).

USG-guided core biopsy was done, and histopathology confirmed a nephroblastoma. Diagnosis of hepatic relapse of WT was, therefore, made. 

She received chemotherapy using the SIOP 2001 stage IV high-risk protocol with alternating courses of Carboplatin, Etoposide, Cyclophosphamide, and Doxorubicin. After five courses of chemotherapy, repeat imaging showed about 50% reduction in the size of the hepatic lesion. She subsequently underwent partial hepatectomy with excision of the hepatic lesion. There was no gross residual. Histopathology, however, showed that the tumor reached the margin at certain areas. The tumor had similar characteristics to her initial renal WT (epithelial type) and showed minimal necrosis. Post-operative imaging done showed a hypodense subcapsular collection in the right lobe with areas of subtle biliary dilatation in the inferior right lobe segments.

She has completed both adjuvant radiotherapy (19.8 Gy to the whole liver in 11 fractions at 1.8 Gy per fraction) and adjuvant chemotherapy. Post-treatment CT scan showed no residual mass with resolution of the previously noted biliary dilatation. She is currently being followed up with three-monthly abdominal ultrasound scans.

Case 4 

An eight-year-old female, a known patient with sickle cell disease (HbSS), presented with a month’s history of progressive painful abdominal distension. A baseline CT scan showed a right renal mass with multiple bilateral pulmonary nodules. An USG-guided biopsy, which was performed on account of her atypical age (age >seven years) per unit protocol, was consistent with WT. She was diagnosed with metastatic right WT and received six weeks of neoadjuvant chemotherapy (Vincristine, Actinomycin D, and Doxorubicin). She underwent a right radical nephrectomy, during which there was an iatrogenic capsular rupture of the tumor. Surgical stage was, therefore, III. 

Repeat chest CT scan post-surgery showed persistence of lung nodules, though reduced in number. She was started on adjuvant chemotherapy using the SIOP 2001 stage IV high-risk protocol with alternating courses of Carboplatin, Etoposide, Cyclophosphamide, and Doxorubicin. She received whole lung external beam radiation therapy (EBRT) to a dose of 12 Gy in eight fractions and 14.4 Gy in 18 fractions to the right flank region. During radiotherapy, however, she defaulted to concurrent chemotherapy treatment. 

**Figure 6:**
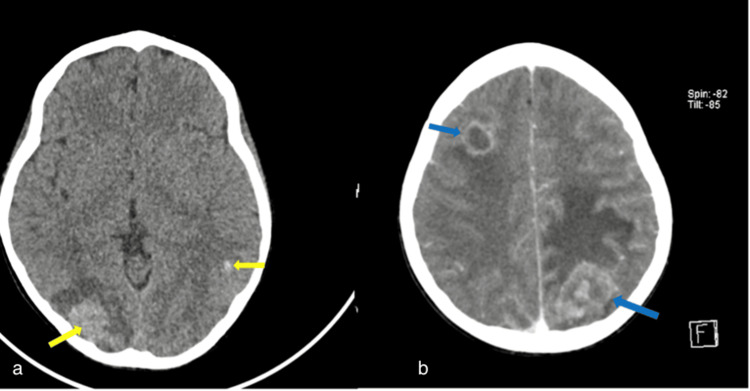
(a) Axial non-contrast head CT scan showing hyperattenuating lesions at the grey, white matter junction (yellow arrows) in the right occipital lobe with surrounding vasogenic edema and a smaller left temporal lobe one (b). The contrast-enhanced axial image showed an anteriorly located right frontal lobe rim-enhancing lesion and a heterogeneously enhancing left parietal lobe lesion with marked vasogenic edema (blue arrows). These were considered metastatic lesions.

After about six weeks of treatment abandonment, she presented to the POU with a day’s history of seizures, vomiting, and persistent headaches, but no altered consciousness. Head CT scan done was suggestive of multiple brain metastases (Figure 6). 

The family was counseled and chemotherapy restarted. She also received craniospinal radiation 25.2 Gy in 14 fractions, with concurrent oral steroid (Dexamethasone). She, however, died at home a few weeks after completion of radiotherapy. 

Table 1 below shows a summary of the key features of each case. 

**Table 1:** Summary of key features of all four cases. N/A, not applicable; SIOP, International Society of Paediatric Oncology

Age (yr)	Sex	Metastasis at initial diagnosis	Metastasis at relapse	Time to metastasis	Histologic classification (SIOP)	Treatment of metastatic disease	Outcome	Follow-up duration if alive (months)
3	M	Bone (jaw)	N/A	N/A	Stromal	Chemotherapy, surgery (marginal excision of mandible)	Alive	8
2	F	No	Bone (vertebrae), spinal canal, lung, mediastinum	1 yr	Epithelial	Palliative care	Palliative care	N/A
13	F	No	Liver	4 yr	Epithelial	Chemotherapy, surgery (partial hepatectomy), radiotherapy	Alive	13
8	F	Lung	Brain	6 weeks	Regressive	Chemotherapy, radiotherapy	Dead	N/A

## Discussion

WT at primary presentation is rare among children above age 10 [1]. In our series, the highest age of presentation of primary disease was eight years. In the UK3 trial, an age greater than four years was found to be associated with an increased risk of distant relapse [12]. All of our patients experienced a distant relapse and only two of them were aged above four years at the time of relapse. 

Even though bone metastasis is extremely rare in WT, two patients in our series (Cases 1 and 2) presented with this; Case 1 as a primary presentation and Case 2 at relapse. Bone metastases are known to occur more commonly in relapsed or progressive disease compared to initial presentation [13]. Even though it was initially in Case 1, he presented about a year after the onset of symptoms, and this may have explained this site of metastasis. 

Bone metastasis in WT appears radiologically as osteolytic bone lesions [7]. In our case, a large lytic mandibular mass was shown on CT imaging.

Jaw metastases in WT have only been documented a few times in literature. The earliest report was published in 1969 and involved a child with multiple metastatic sites, which included the lungs and the mandible [14]. The prevalence of jaw metastases among relapsed non-anaplastic WT cases in the UK3 study was 1.7% [15]. Jaw metastatic WT can be a mimic of more common malignancies such as Burkitt lymphoma.

Spinal metastasis of WT is even rarer. In a review of 718 cases registered in the UKW3 trial, out of the 115 non-anaplastic cases that relapsed, only three cases had bone metastases and none of these involved the spine [15]. In a much earlier study, which reviewed data from the Oxford Survey of Childhood Cancer, however, the most common site of bone metastases was the spine [16]. All reported cases of spinal metastasis occurred at relapse. Case 2 presented with tumor recurrence at multiple metastatic sites, which included multiple vertebrae but also the spinal canal, lungs, and posterior mediastinum. 

Prognosis in bone metastases has been reported to be poor [7]. In a review of patients from the National Wilms Tumor Study database, the five-year overall survival rate for patients with bone metastasis was 14.3% [13]. This was, however, a significant improvement over survival rates reported in the 1980s, where none of the patients survived [16]. Bone metastases at primary diagnosis, however, portend a better outcome compared to when it is present at relapse or as a progressive disease. This is believed to be due to the high propensity of tumor cells being chemotherapy-resistant at relapse compared to primary disease [13].

Prevalence of anaplasia has been found to be much higher in WT with bone metastases (32%) compared with patients with WT in general (6-10%) and most likely is a contributory factor in the poor outcomes reported [13]. The histology of both patients in our series was, however, non-anaplastic. 

Spinal cord compression in WT is also a rare occurrence. It is more common in other malignancies such as neuroblastoma and Ewings sarcoma. When it occurs in WT, it usually involves bony metastases to the vertebrae or intradural or extradural spread, usually in the setting of disseminated disease with multiple metastatic sites [17,18]. This was the pattern demonstrated in our case. It can also occur as a direct spread of a primary tumor, which often occurs in the case of an extrarenal WT [17]. Treatment recommended for spinal compression associated with WT is corticosteroids and immediate commencement of chemotherapy [17]. This option was not explored in Case 2 due to the delay in presentation after onset of the paraparesis and the extent of the disease. 

The presence of brain metastases in WT has been noted to be usually associated with coexisting or preceding metastases to the lung. It has been hypothesized that brain metastasis is most likely tumor emboli, which spread from the lung through a hematogenous route [19]. Case 4 initially presented with pulmonary metastasis, supporting this theory. Clinical features of brain metastases in WT include seizures, headaches, hemiparesis, and decreased consciousness [20,21]. Our patient presented with headaches and seizures. Treatment modalities for brain metastases in WT include surgical excision, chemotherapy, and radiotherapy. The decision on the best treatment modality should be made based on several factors including the number of brain lesions [19]. In our case, surgery was not explored upfront due to the patient presenting with multiple brain lesions. She, however, received both chemotherapy and radiotherapy. Solitary brain metastases are known to have a better prognosis compared to patients presenting with multiple lesions and this may explain why our patient succumbed despite treatment [21].

Most relapses (95%) of WT occur early, within two years after completion of treatment [22]. Relapse after two years even though rare, would usually still occur within five years after initial diagnosis. In a review of 635 children enrolled in the UK3 trial, after a median follow-up of 10 years, none of the patients experienced a first relapse more than four years and one month after initial diagnosis [12]. The term late recurrence generally refers to relapses occurring more than five years after initial diagnosis [23]. Our patient in Case 3 presented four years and eight months after the initial diagnosis. 

Late recurrences, which occur in sites other than the contralateral kidney, are thought to be due to reactivation of cells persisting from the primary tumor, which had hitherto been in a quiescent state. Puberty has been hypothesized to be a possible reactivating stimulus as many late recurrences occur in adolescence [23]. In our series, Case 3 presented at the onset of puberty and hormonal factors may have been a contributory factor to reactivation. The tumor specimen was, however, not studied for the presence of progestin and/or estrogen receptors to confirm this hypothesis [23].

The most frequent site of late relapse is the abdomen. Isolated liver metastasis at relapse is, however, uncommon. In the UK3 study, out of the 115 non-anaplastic cases who experienced a relapse, four had combined lung and liver metastases but there were no recorded cases of isolated liver metastasis [15].

Late recurrences often show the same histologic features as the original tumor [24]. Our patient’s (Case 3) tumor showed predominant epithelial histology both at relapse and at initial diagnosis. This finding also supports the theory of reactivation of persistent dormant tumor cells irrespective of the lag time between initial presentation and recurrence. 

## Conclusions

WT can present with rare sites and patterns of relapse. A high index of suspicion is required to recognize unusual metastatic sites at primary presentation. Metastatic WT, presenting either at diagnosis or relapse, even though generally associated with a poorer prognosis, can still be successfully treated through a multidisciplinary team approach even in a resource-limited setting. Further studies are needed to explore the molecular and genetic basis for unusual metastases in WT.
